# The Glucose Control Resistance Scale

**DOI:** 10.4274/jcrpe.galenos.2018.2018.0164

**Published:** 2019-05-28

**Authors:** Maria-Eleni Nikita, Helen M. Hendy, Keith E. Williams, Paul L. Mueller

**Affiliations:** 1 University of Maryland School of Medicine, Maryland, United States; 2 Penn State University, Schuylkill Campus, Department of Psychology, Pennsylvania, United States; 3 Penn State Hershey Medical Center, Department of Pediatrics, Pennsylvania, United States

**Keywords:** Adolescent beliefs, type 1 diabetes, family conflict, resistance, non-compliance

## Abstract

**Objective:**

While past research found family conflict, disordered eating, body image concerns and anxious self-doubts may affect adolescent diabetic glucose control, available measures of adherence mainly focus on management tasks. The current study aimed to combine measures of emotional distress and beliefs with decisions concerning management in a new measure of resistance to treatment adherence: the 12-item Glucose Control Resistance Scale (GCRS).

**Methods:**

Participants included 135 adolescents and their parents from a pediatric diabetes clinic. Family conflict, body image concerns, anxious self-doubts and glucose control resistance were assessed.

**Results:**

Factor analysis identified 12 items, with loadings of ≥0.40, which were used to form the GCRS. The scale had adequate reliability and there was a significant correlation between child and parent GCRS scores. One factor, family conflict, was significantly related to hemoglobin A1c (HbA1c) levels, but a set of four factors explained a total of 12% of the variance in HbA1c levels. Of the demographic variables considered (gender, number of parents at home, age, body mass index z-score), only gender was significantly associated with adolescent perceptions of family conflict.

**Conclusion:**

The GCRS may allow diabetic care teams to better understand the origin of family conflict perceptions and the motivational beliefs that modify behavior and contribute to independent self-management and glucose control. Each question was designed to be meaningful in interventions by addressing common items of resistance to adherence and impulsive management decisions. The GCRS may be used by providers as an initial short screening survey on an annual or semi-annual basis.

**What is already known on this topic?**

Adolescents with diabetes have more problems with adherence than any other pediatric age group. Previous research has shown that multiple factors, including family conflict, disordered eating and anxiety, are related to adherence.

**What this study adds?**

The Glucose Control Resistance Scale (GCRS) is a measure of adolescent adherence to treatment that may allow diabetic care teams to better understand the origin of family conflict perceptions and the motivational beliefs that modify behavior and contribute to independent self-management and glucose control. Each question was designed to be meaningful in interventions by addressing common items of resistance to adherence and impulsive management decisions. The GCRS may be used by providers as an initial short screening survey on an annual or semi-annual basis.

## Introduction

It is widely accepted that uncontrolled diabetes is associated with increased risk for morbidity and mortality. Current management, based on compliance with treatment recommendations, is often frustrating for patients, their families and providers (1,2). Glucose control can be especially difficult for adolescents due to typical adolescent challenges such as defiance or resistance and the effect this behavior has on their decisions and beliefs regarding diabetes and its treatment. Available screening questionnaires focus on management tasks but fail to link and adapt these tasks to adolescent psychological challenges. Combining attitudes of defiance or resistance with specific decisions on management can be useful in patient care because, for example, lack of motivation may influence compliance as much as the large number of burdensome management tasks.

Challenges regarding diabetic management may be related to developmental changes. Adolescents are becoming more independent, yet often require parental intervention with diabetic care. In some cases. this leads to increased family conflict which has been found to be associated with poorer glycemic control as measured by higher hemoglobin A1c (HbA1c) levels (3,4,5). Increased sexual interest can influence both weight and body image concerns which have been found to result in higher HbA1c (6,7). Additionally, adolescents often report anxiety and self-doubt which have been associated with worse glucose control (3,8). Other beliefs prevalent in adolescents are a sense of physical “invincibility,” identifying with being independent, being in control and defying or being resistant to authority figures, usually the parents (9). These beliefs, combined with a physiologic lag in cognitive processing, can lead to difficulty with focusing on the future or with accepting the adverse consequences of poor metabolic control. These emotional beliefs may also lead to a feeling that there are no problems with resisting medically-recommended guidelines for glucose control. In many cases of uncontrolled diabetes, disordered beliefs in one of four areas of competence (family conflict, disordered eating, anxiety or resistance) are the cause of non-adherence to treatment recommendations (1,2,7).

Commonly used screening tests of adherence measure management skills directly correlated with glucose control (10,11). These measures have largely removed psychological parameters from the management tasks, despite that the psychological factors may limit adherence and require intervention.

Studies have explored issues related to motivation in adolescents with diabetes. For example, self-efficacy and outcome expectations were found to be related to diabetes self-management adherence and glycemic control (12). Motivation has also been integrated into treatment as a growing number of providers advocate the use of motivational interviewing (13). Some surveys contain distress items generated by the burden of management tasks, such as “feeling overwhelmed by my diabetes regime” (14). Other questionnaires list management tasks. However, intervention in one of these areas may not generalize to the other. Attention to both distress and task is needed for improvement of diabetic self-management.

In our experience, discussing both distress and management in the diabetes outpatient visit with both the patient and their family is useful. This discussion can be initiated by exploring whether the patient not only values the required management tasks, but can also accomplish these tasks. This information can help the provider determine whether the adolescent understands these tasks and how the family can be sufficiently supportive to accomplish these tasks. The intervention process begins with identifying a distressing problem as the starting point from which to develop new problem-solving skills related to the specific stress in question. For example, “It’s too hard to calculate my insulin dose” would lead to exploring easier ways for the individual patient to determine a safe dose.

The purpose of the present study was to develop a scale measuring resistance and impulsive decisions, thus enabling providers to be more effective in dealing with distress and fostering management tasks.

## Methods

The primary sample included adolescents with type 1 diabetes from a hospital-based pediatric diabetes program. All patients had been referred to the diabetes program by primary care providers. To examine parents’ perception of their adolescents’ resistance, parents of a sub-group of the patients were asked to answer the same questions presented to the adolescents.

### Item Development

We observed clinically and hypothesized that patients focus on modifying their behavior much more quickly when a perceived burden or distress is discussed in terms of a specific impulsive management decision as these impulsive behaviors may not be admitted by the patient (5). For example, the patient who makes a statement such as “I fear hypoglycemia, so when I feel low I just eat rather than checking my blood sugar” was not likely to admit that he does not check his blood glucose if he were not emotionally engaged by the memory of fear. The questions also explore the burden of managing diabetes which includes the large number of daily self-control tasks required. In aggregate, this burden may lead to distress and maladaptive management decisions. Patient phrases such as “It’s too hard to calculate my insulin dose, so I guess how much to take” are examples of this type of behavior. All questions in this study addressed adolescents with uncontrolled diabetes despite having completed diabetic education. The questions covered areas of diabetic care including diet, insulin administration, blood testing and hypoglycemia management. Each question contained both a burden and impulsive maladaptive management decision, such as “If I think my blood sugar is high, I decide not to take it”. Most questions contained explicit burden and management decision components. For example in one question distress may be implied by the endorsed decision, such as “I do not eat breakfast”. All questions were reviewed and edited by a team of two pediatric endocrinologists, five diabetes educators (three pediatric diabetic nurses and two pediatric dietitians) and a pediatric psychologist.

### Measurements

Research procedures were approved by the Institutional Review Board at Penn State Hershey (protocol no. 37210EP). Informed consent was obtained from both adolescents and their parents/caregivers prior to completion of the surveys. The primary sample of adolescents with diabetes completed surveys to develop the new Glucose Control Resistance Scale (GCRS), and to compare which of four adolescent beliefs best explained variance in the adolescents’ HbA1c levels (see Table 1 for descriptive statistics). In addition to the initial adolescent and parent surveys, a sub-group of the participants completed items from the GCRS on a second occasion, two to four weeks after the initial completion, for assessment of test-retest reliability.

Adolescents and parents completed a survey to report whether or not (0=no, 1=yes) they agreed with 19 possible beliefs about glucose control resistance. Factor analysis identified a final set of 12 questions included in the new GCRS and the score was calculated as the total number of agreements.

The adolescent’s perception of family conflict was measured with the Diabetes Family Conflict Scale (4). The score for family conflict was calculated as the mean three-point rating for the 19 items, and internal reliability for this measure was adequate as measured by Cronbach’s alpha (α=0.86).

The adolescent’s perception of weight and body image concern was measured with the 16-item Diabetes Eating Problem Survey (15). The score for weight concern was calculated as the mean six-point rating for the 16 items and internal reliability for this measure was adequate (α=0.84).

The adolescents’ anxious self-doubts were measured with the 11-item Anxiety Sensitivity Index (16). The score was calculated as the mean five-point rating for the 11 items and internal reliability for this measure was adequate (α=0.85).

### Statistical Analysis

Exploratory factor analysis was conducted for the 19 beliefs using a principle components approach with the requirement that each item showed a factor loading of 0.40 or higher (see Table 2). Internal reliability in the form of Cronbach’s alpha (α) was calculated for the remaining 12 items. Test-retest reliability was calculated as the Pearson correlation coefficient (r) in a sub-group of adolescents across two occasions, two to four weeks apart.

To examine how well parents perceived beliefs of their adolescents, a Pearson correlation coefficient (r) was calculated between the GCRS score of the adolescent and that of the parent.

Because the four adolescent beliefs (Family Conflict, Weight Concerns, Self Doubt and Resistance) are likely to be inter-correlated, their association with HbA1c must be analyzed with more than bivariate correlations. To examine which beliefs held by adolescents were associated with HbA1c, a multiple regression analysis of the whole cohort of adolescents was conducted with HbA1c values serving as the criterion variable and with their four belief scores serving as possible predictor variables: perceptions of family conflict (DFCS), weight and body image concerns (DEPS), anxious self-doubts (ASI), and glucose control resistance (the new GCRS).

To determine which demographic characteristics [gender, number of parents at home, age, body mass index (BMI) z-score] were associated with beliefs found in the above analyses and were associated with HbA1c, a 2 x 2 ANCOVA examined the belief score as the dependent variable compared across two adolescent genders (male, female) and across two parent conditions (both parents at home, single parent at home), with adolescent age and BMI z-score considered as covariates.

## Results

### Patient Demographics

The patient group consisted of 135 adolescents who had been diagnosed with type 1 diabetes. Of these 51.9% were males and 77.4% lived at home with both parents. Mean age was 15.05±2.35 years; mean BMI z-score was 0.73±0.97 and mean HbA1c was 9.03±1.84%.

The subgroup of patients whose parents (n=127) also answered GCRS questions had the following demographic characteristics: 52.0% of male adolescents; 77.4% with both parents at home. Mean age of the sample was 14.94±2.2 years. Mean BMI z-score was 0.72±0.98 and mean HbA1c 9.04%±1.80.

The subgroup of patients who completed the repeat questions 2-4 weeks later for test-retest reliability assessment had the following characteristics: 29/135 (21.5%) took part, 41.4% were male; 72.4% had both parents at home; mean age was 15.15±1.93 years, mean BMI z-score was 0.60±0.76 and mean HbA1c was 9.39±1.68%. Thirty-five adolescents also completed a follow-up survey following a mean interval of 1.8 years (6 months-4 years).

### Psychometrics of the New GCRS

Exploratory factor analysis revealed a principal component of 12 items showing factor loadings of 0.40 or higher which would be selected to comprise the new GCRS (Table 2). Internal reliability was adequate for both the adolescents’ beliefs (α=0.80) and for their parents’ perceptions of their beliefs (α=0.81). Test-retest reliability was 0.68, near and only slightly lower than the traditionally recommended 0.70 value (r=0.68, p<0.001). Additionally, see Table 3 for bivariate correlations for each pair of the four adolescent beliefs examined in the present study: glucose control resistance, family conflict, weight concern, anxious self-doubt.

### How well Do Parents Understand Their Adolescents’ Glucose Control Resistance?

The Pearson correlation coefficient of the the adolescents’ and parents’ GCRS scores suggests parents had moderately high understanding of their adolescents’ glucose control resistance beliefs (r=0.50, p<0.001).

### Which of Four Adolescent Beliefs are Associated with Glucose Control (HbA1c)?

The multiple regression analysis revealed that of the four adolescent beliefs considered (family conflict, weight concerns, self-doubts, and glucose control resistance), only family conflict was significantly associated with worse (higher) HbA1c levels (r=0.31), with the set of four beliefs explaining a total of 12% of the variance in HbA1c levels (R2=0.12; see Table 4). Although not associated directly with HbA1c, GCRS was highly correlated with family conflict (r=0.54) and with weight/eating concerns (r=0.59). Also associated with family conflict were weight/eating concerns (r=0.71), and anxiety (r=0.40).

### Which Adolescent Demographics are Associated with Their Family Conflict Beliefs?

The 2 x 2 ANCOVA revealed that of the adolescent demographic variables considered (gender, number of parents at home, age, BMI z-score), only gender was significantly associated with adolescent perceptions of family conflict with females reporting more than did males (female mean±SD=0.30±0.30; male mean±SD = 0.21±0.22; see Table 5).

## Discussion

A new GCRS showed strong psychometric characteristics of internal reliability and adequate test-retest reliability, both for the adolescents themselves and their parents. The psychological factor of defiance or “resistance” to recommended glucose control practices has frequently not been the focus of screening surveys. A correlation of GCRS was found with a measure of anxiety and a high correlation with weight concern and family conflict, providing convergent validity with measures important for diabetic self-management. The unique feature of “resistance” and the convergent validity with other scales of diabetic distress suggest that GCRS may be useful as an initial short screening measure in the diabetic follow-up routine on an annual or biannual basis. Items could be addressed in real time at the outpatient visit and, if needed, changes in management could be made, such as referral to a psychologist or other provider, further education, or more intensive follow-up. The items may also present an opportunity for providers to utilize the protective processes of benefit finding, optimism, or adaptive coping with a specific diabetic management task. This approach has been shown to ameliorate family conflict (17).

Another new feature of the present study is comparison of the four areas of competence for their association with HbA1c levels. The four beliefs included perceptions of family conflict, weight and body image concerns, anxious self-doubts, and the concept of glucose control resistance as measured by the newly developed scale, the GCRS described herein. Taken together, these four adolescent beliefs explained 12% of the variance in A1c levels. The scales of individual psychologic parameters (GCRS, weight concern, anxiety) were all moderately or highly correlated with family conflict (Table 3) and family conflict was correlated with HbA1c, explaining 9% of the variability. This suggests reducing family conflict is a critical step in glucose control, and that in working on the reduction of family conflict, it may be important to address the beliefs and decisions of GCRS, weight concerns or anxiety in the adolescent.

Our findings support the reported correlation of family conflict and HbA1c levels (4,18). Poor control by the adolescent generates conflict and any resultant strict authoritarian style of parents may lead to anger or anxiety in the adolescent with worsening glucose control and threatened self-motivation (19).

Both diabetic management and family therapy with the adolescent are challenging but the GCRS may be used for problem-solving in the office or to monitor a patient after referral to a mental health care provider. The most productive focus is how family can help the adolescent improve self-management which then improves feelings of self-sufficiency. The key is to be helpful when needed (authoritative) but not controlling (authoritarian) (20) and the tone of communication is more important than the frequency of talking (20,21). Continued family involvement as the teen shares greater responsibility is recommended (7).

Anxiety which may involve needle phobia or fear of hypoglycemia (22) was found to predict HbA1c one year later (3). Depression was correlated with HbA1c but knowledge of diabetic management was not (23). Symptoms of anxiety and depression are frequently found in the same patient, with anxiety reported even more frequently than depression. Screening for anxiety has been proposed to be an adequate measure for identifying those at risk of depression (24). Diabetic distress was correlated with anxiety and family conflict (25) and supported our findings that GCRS questions of distress and decision were correlated with anxiety and highly correlated with family conflict.

Use of the GCRS may uncover previously unmentioned information that can be utilized in treatment. The set of 12 questions contains several areas of impulsive or maladaptive behavior decisions. One type of behavior in several questions is a lack of action, possibly based on a belief that it is acceptable to deny the problem, defy parents or feel invincible. For example, “If I think my blood sugar is high, I decide not to check it”. Another type of behavior is endorsing “liking” as a justification for not adhering to recommendations, such as “I like to get or buy extra food at school, more than on my meal plan”. This new measure may allow adolescents to become more comfortable in discussion with health care providers, help both patient and provider better understand concerns of the adolescent and thus enable behavioral change. For example, in response to the question “It’s too hard to calculate my dose, so I just guess how much to take,” discussion with the adolescent might include how this decision makes him feel and the benefits he perceives from the belief. Discussion might unveil underlying needs and feelings that, when expressed, can facilitate the discovery of alternative approaches in management that are workable and acceptable to the family.

### Study Limitations and Directions for Future Research

One limitation of the present study was the relatively small sample size of 135 adolescents diagnosed with type 1 diabetes. Also, participants came exclusively from a diabetes clinic in the northeastern region of the United States and demographic information did not include their ethnic identity, sexual orientation or religious affiliation. Future research should include larger and more diverse samples of adolescents to conduct confirmatory factor analyses for the new GCRS and to determine whether family conflict continued to be the adolescent belief most strongly associated with poor glucose control. Future research might focus more specifically on the use of the GCRS in management of family conflict and on which parenting styles (such as permissive, authoritative, authoritarian) improve the adolescent’s motivation for diabetic self-management, internalizing self-sufficiency and quality of life.

## Conclusion

The findings from the present study show that the GCRS is correlated with HbA1c. However the comparison of the four areas of belief demonstrates that family conflict is the most significant predictor of HbA1c. These results may alert clinicians to the importance of addressing family conflict as part of their overall diabetes intervention.

## Figures and Tables

**Table 1:**
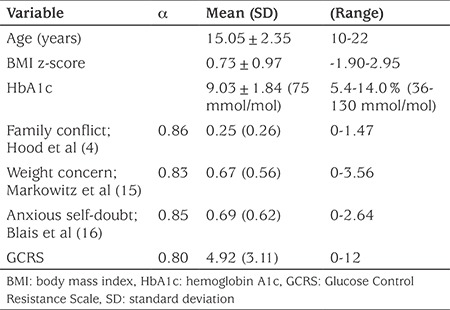
Descriptive demographic data of the 135 participants

**Table 2:**
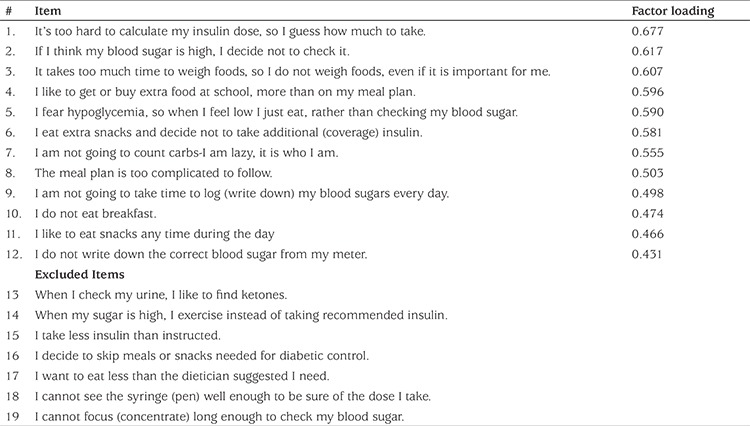
Exploratory factor analysis results for the Glucose Control Resistance Scale

**Table 3:**
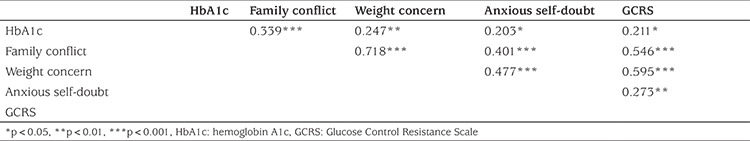
Bivariate correlations between study variables

**Table 4:**
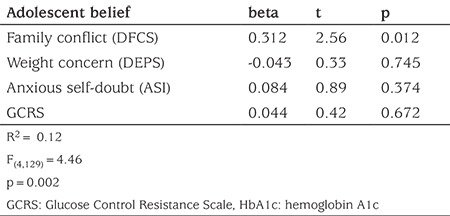
Multiple regression analysis of four adolescent beliefs for their association with glucose control (HbA1c)

**Table 5:**
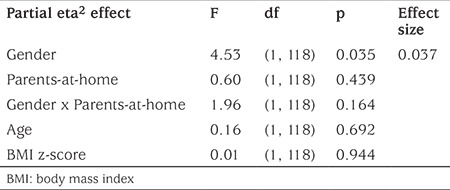
2 x 2 ANCOVA to examine demographics associated with family conflict beliefs in adolescents with type 1 diabetes, comparing cross two genders, two parents-at-home conditions with adolescent age and body mass index z-score as covariates
